# Investigating the morphological and genetic divergence of arctic char (*Salvelinus alpinus)* populations in lakes of arctic Alaska

**DOI:** 10.1002/ece3.7211

**Published:** 2021-03-05

**Authors:** Stephen L. Klobucar, Jessica A. Rick, Elizabeth G. Mandeville, Catherine E. Wagner, Phaedra Budy

**Affiliations:** ^1^ Department of Watershed Sciences and the Ecology Center Utah State University Logan UT USA; ^2^ Department of Botany University of Wyoming Laramie WY USA; ^3^ Program in Ecology University of Wyoming Laramie WY USA; ^4^ Wyoming Cooperative Fish and Wildlife Research Unit Department of Zoology and Physiology University of Wyoming Laramie WY USA; ^5^ U.S. Geological Survey Utah Cooperative Fish and Wildlife Research Unit Logan UT USA; ^6^ Present address: Institute of Arctic Biology University of Alaska Fairbanks Fairbanks AK USA; ^7^ Present address: Department of Integrative Biology University of Guelph Guelph ON Canada

**Keywords:** arctic char, divergence, fish morphology, genomics, lakes, polymorphism

## Abstract

Polymorphism facilitates coexistence of divergent morphs (e.g., phenotypes) of the same species by minimizing intraspecific competition, especially when resources are limiting. Arctic char (*Salvelinus* sp.) are a Holarctic fish often forming morphologically, and sometimes genetically, divergent morphs. In this study, we assessed the morphological and genetic diversity and divergence of 263 individuals from seven populations of arctic char with varying length‐frequency distributions across two distinct groups of lakes in northern Alaska. Despite close geographic proximity, each lake group occurs on landscapes with different glacial ages and surface water connectivity, and thus was likely colonized by fishes at different times. Across lakes, a continuum of physical (e.g., lake area, maximum depth) and biological characteristics (e.g., primary productivity, fish density) exists, likely contributing to characteristics of present‐day char populations. Although some lakes exhibit bimodal size distributions, using model‐based clustering of morphometric traits corrected for allometry, we did not detect morphological differences within and across char populations. Genomic analyses using 15,934 SNPs obtained from genotyping by sequencing demonstrated differences among lake groups related to historical biogeography, but within lake groups and within individual lakes, genetic differentiation was not related to total body length. We used PERMANOVA to identify environmental and biological factors related to observed char size structure. Significant predictors included water transparency (i.e., a primary productivity proxy), char density (fish·ha^‐1^), and lake group. Larger char occurred in lakes with greater primary production and lower char densities, suggesting less intraspecific competition and resource limitation. Thus, char populations in more productive and connected lakes may prove more stable to environmental changes, relative to food‐limited and closed lakes, if lake productivity increases concomitantly. Our findings provide some of the first descriptions of genomic characteristics of char populations in arctic Alaska, and offer important consideration for the persistence of these populations for subsistence and conservation.

## Graphical abstract

In this study, we described the morphological and genetic diversity of seven populations of arctic char with varying length‐frequency distributions across two distinct groups of lakes in northern Alaska. Although some lakes exhibit multimodal size distributions, using model‐based clustering of morphometric traits corrected for allometry, we did not detect morphological differences within and across char populations. Genetic analyses demonstrated differences among lake groups, but within lake groups and within individual lakes, genetic differentiation was not related to total body length.

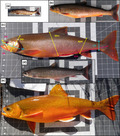

## 1 INTRODUCTION

Complex selection pressures influenced by environmental factors and resource availability can determine the adaptive potential and persistence of populations (Reznick & Ghalambor, 2001). Phenotypic plasticity allows for morphological and physiological responses to spatial and temporal variation in the environment, and thus, species’ evolution may stem in important ways from plasticity (Agrawal, 2001; Schulte et al., 2011). However, less abundant populations, especially in smaller ecosystems (e.g., small, isolated lakes), may have limited adaptive potential due to genetic bottlenecks that have reduced genetic diversity. In addition, other intrinsic factors (e.g., dispersal capabilities) can limit the fitness of different phenotypes of the same species (e.g., “morphs”; DeWitta et al., 1998; Willi et al., 2006) and further can regulate community structure across systems with variable landscape connectivity (e.g., Hershey et al., 1999).

Considerable differences in morphology, life history, and behavior of fishes often result from differences in resource and habitat‐related selection (e.g., Power et al., 2005), and these differences may be even more profound within isolated lakes (Skúlason & Smith, 1995). Resource polymorphism can facilitate the coexistence of morphs of the same species in the same environment, especially when resources are limiting (Svanbäck & Persson, 2004). This polymorphism, which may arise from genetic factors or from phenotypic plasticity (Andersson, 2003), can aid in maintaining genetic diversity within populations. However, in many cases, phenotypic divergence underlying phenotypic polymorphism may, at least initially, result from plasticity rather than genetic divergence (Schluter, 2000). For example, in temperate lakes, pumpkinseed sunfish (*Lepomis gibbosus*) can exhibit high degrees of intraspecific variation in jaw morphology based on the availability of a primary prey (gastropods), yet common garden experiments show these differences are driven by plasticity instead of rapid evolution (Mittelbach et al., 1999; Robinson & Wilson, 1996). In some cases, polymorphisms that initially result from plasticity may lead to genetic divergence and differentiation through genetic accommodation, and the situations under which this may happen have received considerable interest (Bock et al., 2018; West‐Eberhard, 2005; Wund et al., 2008).

In high latitudes, arctic char (*Salvelinus alpinus*) exhibit widespread polymorphism across their range and within lakes (Jonsson & Jonsson, 2001). In fact, Klemetsen (2013) regarded arctic char as “the most variable vertebrate on Earth.” Within‐lake divergence of morphs based on habitat (e.g., littoral, pelagic, profundal) and diet (e.g., planktivorous, piscivorous) acts to limit intraspecific competition within char populations, and up to six distinct char morphs can occur within the same lake (Doenz et al., 2019; Jonsson & Jonsson, 2001; Klemetsen, 2010). However, the presence of distinct morphs, and the exact number of morphs, is highly variable from lake to lake which could be attributed to abiotic (e.g., ecosystem size, habitat availability), biotic (e.g., prey availability), and evolutionary (e.g., genetic diversity) factors, alone or in combination. Overall ecosystem sizes, including lake depth, surface area, and volume, are positively correlated with the degree of habitat segregation and, accordingly, polymorphism, in lakes in Scotland and Ireland (Recknagel et al., 2017). In addition, interactions with other species can directly or indirectly affect char trophic dynamics and survival (Eloranta et al., 2013). Water temperature can also influence overall resource availability and rate of consumption which thereby influence growth rates (Hindar & Jonsson, 1993; Rikardsen et al., 2000). With such dependence on biotic and abiotic factors, the development or persistence of a particular morph can be highly variable and unpredictable as environmental conditions change, particularly when these phenotypes are plastic rather than due to genetically differentiated ecotypes. Understanding when ecomorph divergence is underlain by plasticity and when it is genetically determined is a key unanswered question in the study of arctic char populations.

While polymorphism among sympatric char morphs is common across their range, some studies have found significant genetic differences among these divergent ecotypes (e.g., Gislason et al., 1999; May‐McNally et al., 2015; Skúlason et al., 1996), while others attribute morphs to plasticity (e.g., Andersson, 2003; Klemetsen, 2010) . Thus, it is important to understand whether polymorphic char types are genetically distinct and reproductively isolated. Genetic divergence among ecomorphs of char can arise either from differences in functional traits related to feeding ecology (e.g., jaw morphology or fin anatomy; Arbour et al., 2011; Bryce et al., 2016) or from differences in life histories (e.g., May‐McNally et al., 2015; Skúlason et al., 1996). The magnitude and consequences of genetic differences can vary among ecomorph types that diverge in sympatry and allopatry, with sympatric pairs potentially demonstrating higher levels of genetic differentiation than allopatric pairs (Praebel et al., 2016). In addition, genetic differences among ecomorphs can lead to divergent life histories (Praebel et al., 2016). Genetically based differences in allometry can have functional consequences related to behavior and life history, such as predator avoidance (e.g., Knutsdotter Simonsen et al., 2017), suggesting these differences can also contribute to differential fitness among ecomorphs. The high diversity of arctic char populations and associated trophic dynamics (e.g., Klobucar et al., 2018; Klobucar & Budy, 2020) make them ideal models for identifying the underlying genetic basis for these phenotypes in nature, which may help to predict potential evolutionary pathways under changing environmental conditions (Elmer, 2016; Violle et al., 2014).

Postglacial lakes are often viewed as ideal systems to study adaptive processes such as the origins and maintenance of resource polymorphism (e.g., Schluter, 1996; Snorrason & Skúlason, 2004) due to relatively low species diversity and productivity, and frequent high habitat segregation between littoral and pelagic morphs (e.g., Klemetsen, 2010; Pielou, 2008). In postglacial lakes, colonization and adaptation have occurred relatively recently, as recent as 10,000 years ago (e.g., Skúlason et al., 1989). The age of the glacial landscape affects physical, chemical, and biological characteristics that may underpin morphological segregation of arctic char populations across the landscape (Hershey et al., 1999; Luecke et al., 2014). For example, lakes at higher elevations typically have decreased surface area, steeper shorelines, and may not have clearly segregated habitat zones within a lake. These lakes are also less connected to surrounding surface waters, creating barriers to gene flow, and populations of char in these lakes are more isolated than populations in lakes at lower elevation and with more surface water connections. However, as the Arctic continues to warm, loss of surface water connectivity between lakes (e.g., seasonal drying of streams) or reduced availability of suitable habitat due to the combination of increased water temperatures and decreased levels of dissolved oxygen in lakes may disrupt access to habitats that create or maintain char polymorphism (Hobbie & Kling, 2014).

In this study, we examined potential morphological and genomic differences between arctic char populations within and across two geographically close, but otherwise contrasting, lake groups in the foothills of the Brooks Range, Alaska. These lakes vary in their abiotic (e.g., surface water connectivity, surface area) and biotic characteristics (e.g., species richness, primary production), and we used these gradients to potentially explain the divergence of arctic char populations and their size structures. First, we tested for morphological differences between the arctic char populations of varying size structures, and determined the factors potentially contributing to char morphological divergence. Secondly, we used a genotyping‐by‐sequencing approach to determine the genetic diversity and extent of genetic differentiation across morphs and lake groups. Ultimately, our findings contribute to our understanding of how abiotic and biotic factors can structure arctic char populations and provide some of the first descriptions of char morphological and genetic divergence in northern Alaska.

## 2 METHODS

### 2.1 Study site

Our research was conducted in lakes near Toolik Field Station (68°37.796′N, 149°35.834′W), home of the Arctic Long‐Term Ecological Research project (http://arc‐lter.ecosystems.mbl.edu/), in the northern foothills of the Brooks Mountain Range, Alaska. Lakes in this region were formed by glaciers over three periods approximately 12–25 ka, 53–100 ka, and 250–300 ka in age (Hamilton, 2003). Generally, the lakes are shallow (maximum depths of 3–30 m) and ultra‐oligotrophic, or low in productivity (chlorophyll‐α concentrations < 5 µg/L; Kling et al., 1992). Fish community composition is broadly determined by landscape factors (e.g., lake depth, surface water connectivity), but overall, fish species richness is low, with maximum species richness of five species (Hershey et al., 1999, 2006).

**FIGURE 1:**
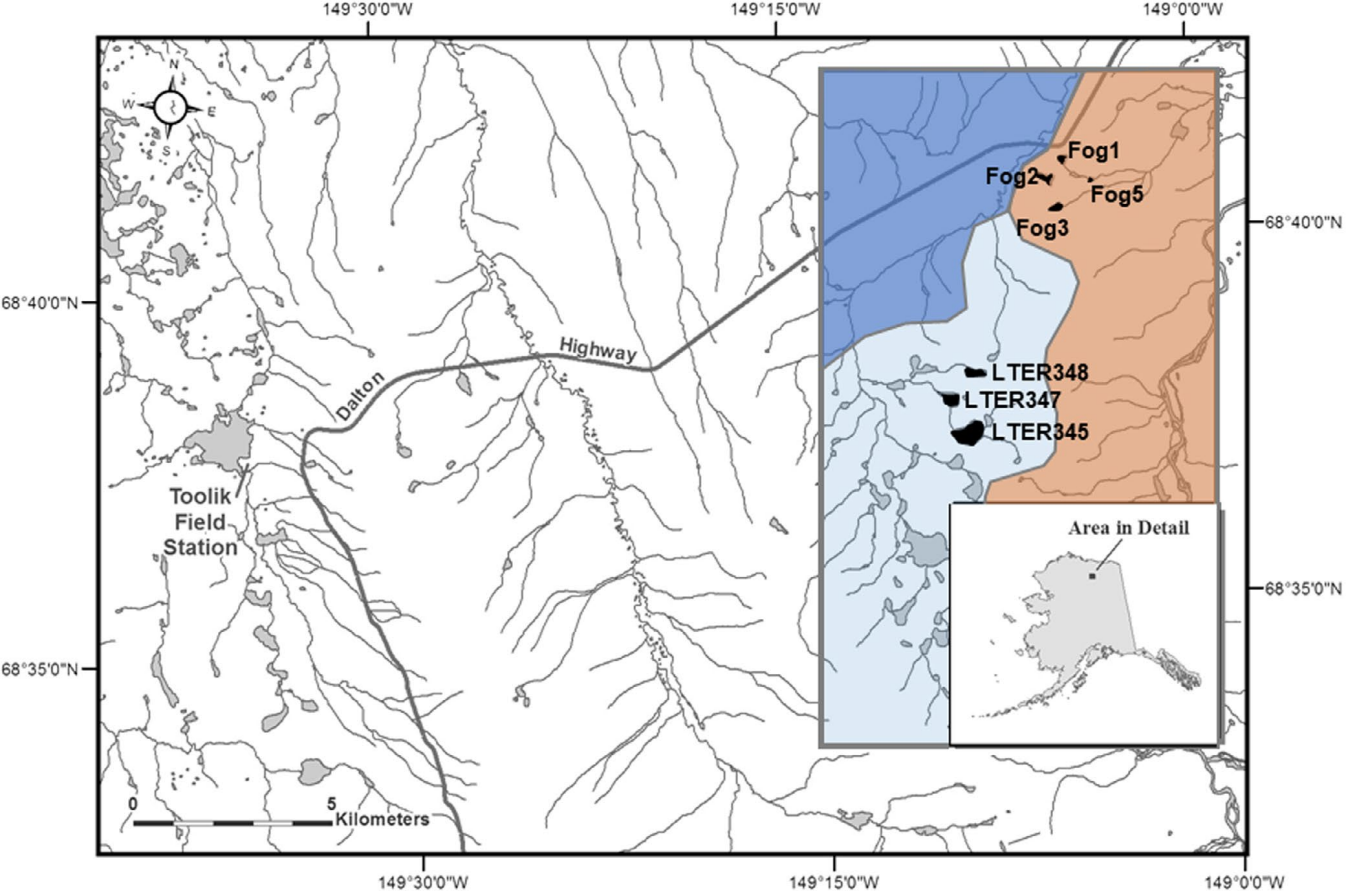
Map of the study area in northern Alaska. Colored inset represents different glacial landscape ages (dark blue = Sagavanirktok River glaciation, Middle Pleistocene, >125,000 years before present; light blue = Itkillik Phase I glaciation, Late Pleistocene, >53,000 years before present; orange = Itkillik Phase II glaciation, Late Pleistocene, 11,500–25,000 years before present) adapted from Hamilton, 2003

We measured arctic char morphology from seven lakes in two distinct lake groups (Figure 1; Table 1). One group of lakes (*n* = 4; the Fog lakes) lacks surface water connectivity (“closed”) and contains arctic char as the only apex predator. The other series of lakes (*n* = 3; the LTER lakes) are connected by surface water (e.g., inlet and outlet steams, “leaky”) and contain arctic char as well as arctic grayling (*Thymallus arcticus*), lake trout (*Salvelinus namaycush*), and burbot (*Lota lota*) as potential competing predators. All of the Fog and LTER lakes also contain slimy sculpin (*Cottus cognatus*) as a potential prey fish. While these lake groups are located in close proximity (~5 km), they are situated on different glacial landscapes (Fog lakes = Itkillik II, 12–25 ka; LTER lakes = Itkillik I > 53 ka). In combination with contrasting connectivity, this suggests different colonization periods and different potential for historic gene flow. The LTER lakes are found in a headwater subbasin of the Sagavanirktok River drainage whereas the Fog lakes are located in the main drainage of Sagavanirktok. All study lakes thermally stratify during summer months.

**TABLE 1:** Lake morphometry and fish community composition (AC = arctic char, AG = arctic grayling, BT = burbot, LT = lake trout, SS = slimy sculpin) for study lakes. Lake area and volume < 3 m are proportions of the total area or volume. AC abundance is population estimates from Klobucar et al., 2017 (Fog lakes) and modified Schnabel estimations from mark–recapture (LTER lakes)

Group	Lake	Latitude (°N)	Longitude (°W)	Surface area (ha)	Lake volume (m^3^.10^5^)	Max depth (m)	Mean depth (m)	Lake area < 3m	Lake volume < 3m	Secchi depth (m)	Fish community	AC abundance	AC density (fish·ha^−1^)
Fog	Fog1	68.684	149.082	3.5	2.9	19.7	8.4	0.33	0.29	4.9	AC, SS	448 (290–693)	128
	Fog2	68.679	149.091	5.9	4.4	19.8	7.8	0.21	0.34	7.1	AC, SS	163 (105–288)	27
	Fog3	68.673	149.088	3.9	3.1	21.0	7.6	0.30	0.31	6.0	AC, SS	666 (477–1,073)	170
	Fog5	68.678	149.065	0.7	0.3	9.9	3.5	0.52	0.61	5.0	AC, SS	75 (55–119)	107
LTER	LTER345	68.623	149.151	30.7	38.2	28.6	12.3	0.16	0.22	1.5	AC, AG, LT, SS	277 (177–540)	9
	LTER347	68.625	149.139	13.5	7.6	17.6	5.6	0.28	0.45	1.8	AC, AG, LT, SS	73 (40–196)	5
	LTER348	68.641	149.127	5.7	1.9	9.6	3.2	0.56	0.70	3.7	AC, BT, SS	331 (227–563)	58

### 2.2 Arctic char morphometric traits and growth

We sampled arctic char in 2016 (May–September) and 2017 (May) via gill nets and hook‐and‐line sampling. We used eight‐panel, experimental benthic gill nets (gill net mesh size range = 18–64 mm; Lester et al., 2009) set perpendicular to shore on the lake bottom, which extended from the littoral zone to bottom depths in open water areas, and checked nets every half hour to minimize mortalities. We conducted hook‐and‐line sampling alone through the ice (May) and concurrently with gill nets during open water periods. We used a mix of hook‐and‐line methods (e.g., lure size), in addition to experimental gill nets, to sample char across sizes classes. We sampled all lakes at similar time periods and used same sampling methods at each lake. Therefore, we are confident we sampled across all fish greater than approximately 115 mm (but see Finstad and Berg 2004). For example, hook‐and‐line sampling was conducted both in littoral and in open water habitats throughout the water column. For each arctic char captured, we measured, weighed, and then photodocumented the fish on a grid board for later trait measurement. We placed each fish flat, oriented head to the left, and photographed the fish from approximately 60 cm directly above the fish prior to releasing the char.

**FIGURE 2:**
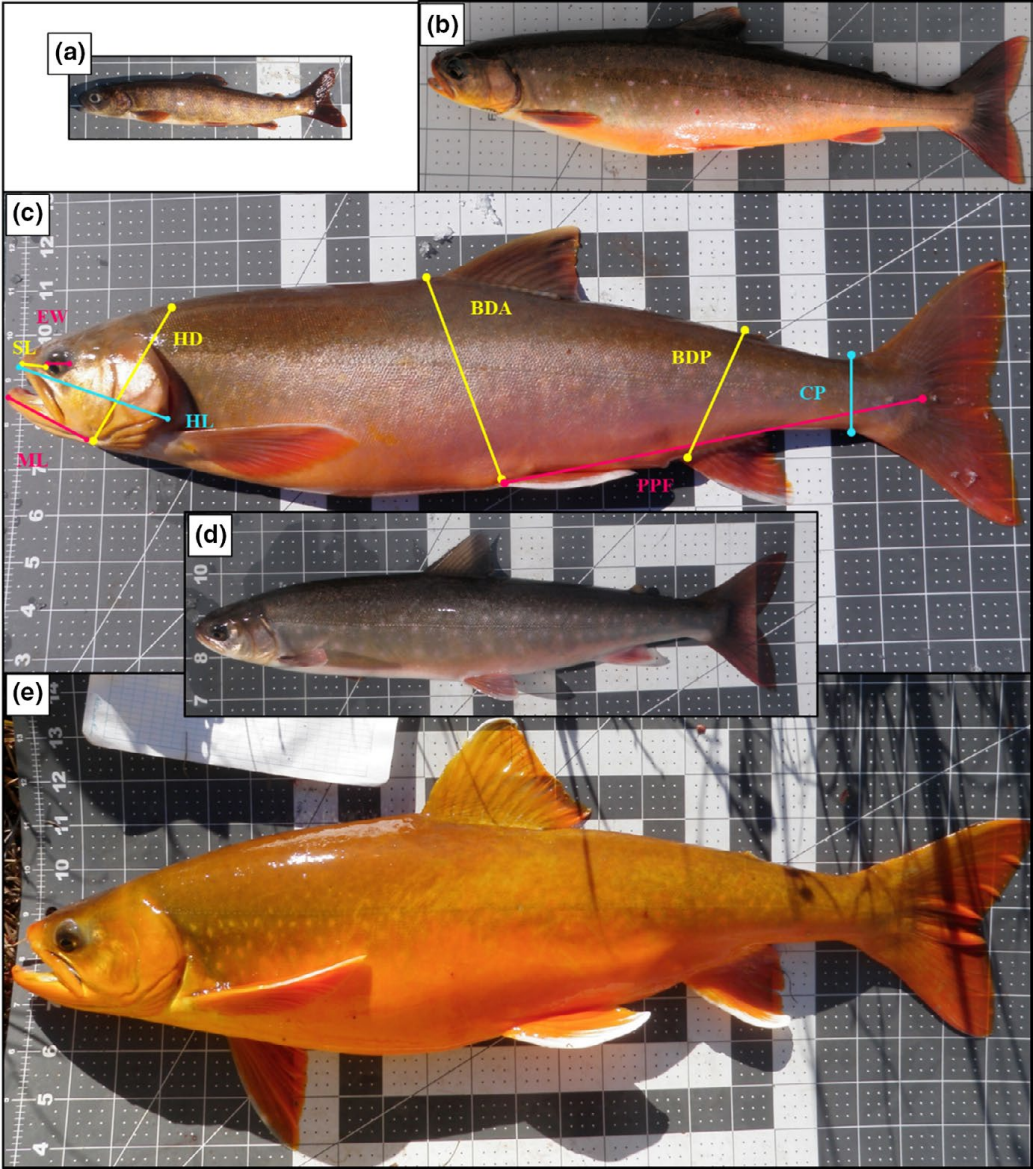
Examples of arctic char found in the “closed” Fog and “leaky” LTER lakes near Toolik Field Station, Alaska. Fish are scaled to the largest fish. (a) “Small” char from Lake Fog3 aged at 9 years old when captured (TL = 160 mm). (b) “Medium” char from Lake Fog3 aged at 11 years old when captured (TL = 350 mm). (c) “Large” char from Lake LTER348 (TL = 578 mm) with colored lines provided as an illustration of the nine morphometric measurements made on each char in this study: snout length (SL), eye width (EW), maxilla length (ML), head depth (HD), head length (HL), body depth posterior (BDP), body depth anterior (BDA), postpelvic fin length (PPF), and caudal peduncle depth (CP); D) “Medium” char from Lake LTER348 (TL = 337 mm); and E) “Large” char from Lake LTER348 (TL = 587 mm)

We subsequently used photographs to make morphometric measurements (mm) including: snout length (SL), eye width (EW), maxilla length (ML), head depth (HD), head length (HL), body depth posterior (BDP), body depth anterior (BDA), postpelvic fin length (PPF), and caudal peduncle depth (CP), using the software program ImageJ (e.g., Skoglund et al., 2015; Figure 2). To account for allometric size differences, we first log‐transformed measurements to reduce heterogeneity in variance and then size‐adjusted our measurement using an allometric growth formula (e.g., Senar et al., 1994):

$$\log^{10}Y_{i}=\log^{10}M_{i}+b\left(\log^{10}L_{m}‐\log^{10}L_{i}\right).$$

where *Y_i_* is the size‐adjusted trait value, *M_i_* is the measured trait value, *L_i_* is the measured total length, *b* is the slope of the log‐transformed measured trait (*log^10^M_i_*) against log‐transformed total length (*log^10^L_i_*), and *L_m_* is the mean total length for all fish (e.g., all char for comparisons across lakes, all char within a lake for within‐lake comparisons).

We collected otoliths from a subset of arctic char (e.g., opportunistically and incidental mortalities) captured during our gill net and hook‐and‐line sampling (*n* = 18 in Fog lakes, *n* = 18 in LTER lakes) to further examine growth and size at age of arctic char across the study systems. Due to relatively small char population abundances in our study lakes, we limited opportunistic otolith collection to sample otoliths from size classes of char not already collected via incidental mortalities (e.g., small char in Lake Fog3). Otoliths were mounted with glue on a slide and sanded to expose annual growth rings. We measured annual growth along a radius from the origin to the edge perpendicular to the growth rings and back‐calculated length at age using the biological intercept method (Campana, 1990). We calculated the biological intercept by using an observed linear relationship of log‐transformed annual growth and otolith age for the five youngest fish collected and used an average length at hatch of 17 mm (Nordeng, 1983).

### 2.3 Statistical analyses

**FIGURE 3:**
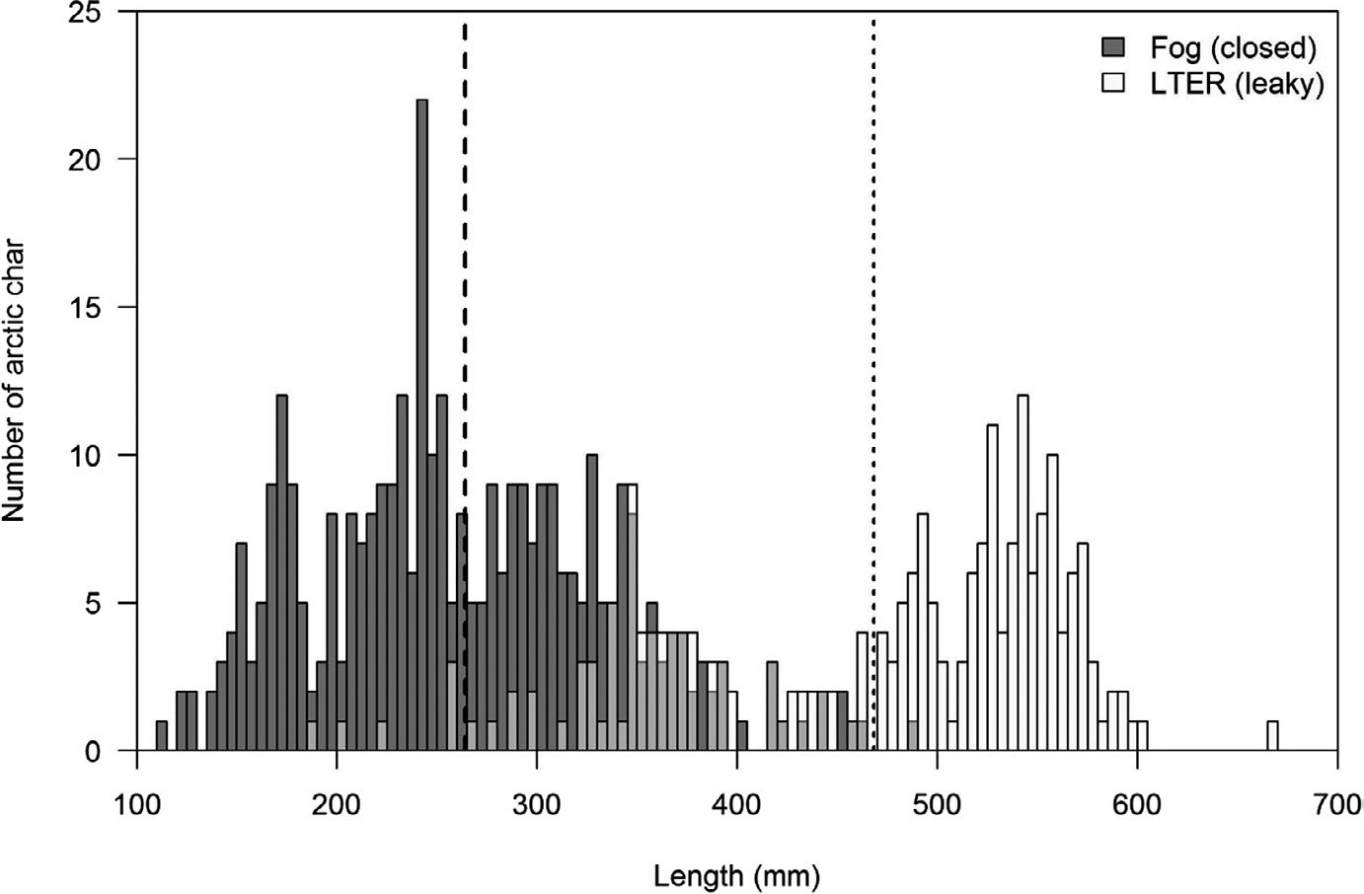
Length‐frequency histogram of arctic char captured in the Fog and LTER lakes 2014–2017. Middle gray represents the overlap between Fog (dark gray) and LTER (white) catches

To test for morphological differences, we performed model‐based clustering on the size‐adjusted trait measurements using the “mclust” package (version 5.4; Scrucca et al., 2016) and selected the number of classifications based on clustering that maximized Bayesian information criterion (BIC) with ΔBIC > 3 from the next closest number of clusters. We expected distinct char morphs to have distinct body and head shapes that best suit the ecology of a given morph, and thus, these differences would not explained by allometry alone (e.g., Jonsson & Jonsson, 2001; Skúlason et al., 1989). Our preliminary analyses indicated different arctic char length distributions within and between the Fog and LTER lake groups (Figure 3), suggesting morphological differences. Initial size‐adjusted clustering analyses did not reveal morphological differentiation. We therefore reanalyzed the trait measurements without size adjustments (e.g., raw trait measurements) to classify the observed char size class distributions across all lakes.

Following these cluster models, we used PERMANOVA analyses (adonis.II) in the “RVAideMemoire” package (version 0.9‐69; Herve, 2018) to determine the abiotic and biotic factors that may determine either: (a) the potential drivers of distinct arctic char morphs; or (b) the potential drivers of arctic char size classes and growth patterns. We considered PERMANOVA predictors significant at α < 0.05 and included abiotic factors of: maximum lake depth (m), mean lake depth (m), lake surface area (ha), lake volume (m^3^· 10^5^); and biotic factors of: char abundance, fish density (ind/ha), and Secchi depth (m; as an index of primary production; Table 1, see also http://arc‐lter.ecosystems.mbl.edu/ for standard methodology and further lake information). As each of the LTER lakes contains other apex fishes and the Fog lakes contain only char as the apex predator, we first tested for a potential effect of lake group, which incorporates both lake connectivity and fish species richness, as a categorical predictor of arctic char size structure. We also tested the abiotic and biotic factors above, singularly and additively, to construct the best fitting model with significant predictors and that minimized residual sums of squares. All statistical analyses of char morphological data were conducted in R version 3.5.0 (R Core Team, 2018).

### 2.4 Genomic analyses

For each arctic char we photodocumented for the two lake groups, we also collected a fin clip from the anal fin for genomic analyses. Fin clips were stored in 95% ethanol until DNA extraction. We generated a genotyping‐by‐sequencing dataset to determine whether lakes with bimodal size distributions of fish exhibited genetic divergence between putative ecotypes (Elshire et al., 2011; Parchman et al., 2012). We extracted DNA from archived fin clips using a QIAcube DNA extraction robot using DNEasy Blood &Tissue extraction (Qiagen, Inc.), according to the manufacturer's instructions. We then prepared reduced‐representation genomic libraries using a protocol (Parchman et al., 2012) that starts by digesting DNA with two restriction enzymes, MseI and EcoRI. Following the restriction digest, we ligated unique nucleotide barcodes to each individual's DNA. To increase the template for sequencing, we then amplified barcoded DNA using PCR. We multiplexed 192 individuals per lane of sequencing. Prior to sequencing at the University of Texas Genome Sequencing and Analysis Facility, the genomic library was size‐selected using BluePippin (Sage Science) to retain only fragments 250–400 base pairs in length. The two genomic libraries were sequenced on one lane each of an Illumina Hiseq 4,000 (SE, 1 × 150).

All analyses of genomic data requiring high‐performance computing were conducted on the University of Wyoming's Mount Moran IBM SystemXcluster (Advanced Research Computing Center, 2012) and Teton Intel x86_64 cluster (Advanced Research Computing Center, 2018), and R analyses used version 3.5.3 (R Core Team, 2019). Prior to population genomic analyses, we completed several bioinformatics steps necessary for processing data, similar to Mandeville et al., (2017) and Underwood et al. (2016). First, we filtered common contaminants and unwanted sequences (PhiX, E. coli, and leftover barcodes, primers, and adaptors from library preparation) from our data using Bowtie2 (Langmead & Salzberg, 2012). We then matched sequences to individual fish using a custom barcode parsing Perl script. All data were assembled to the Atlantic salmon genome (Lien et al., 2016) using the bwa mem algorithm (v0.7.17; Li, 2013; Li & Durbin, 2009). We used the Atlantic salmon genome because it is a high‐quality reference genome from a relatively closely related species. After assembly, we removed individuals from the dataset who had fewer than 10,000 assembled reads. We then identified variable sites (i.e., single nucleotide polymorphisms; SNPs) in the assembly using SAMtools and BCFtools (v1.8; Li, 2011; Li et al., 2009). We filtered those SNPs by minor allele frequency and amount of missing data using VCFtools (v0.1.14; Danecek et al., 2011) to allow no more than 50% missing data and retained only SNPs with minor allele frequency greater than 0.01 to decrease the risk of including of SNPs that represent sequencing error, and to emphasize major axes of genetic differentiation. We also analyzed a dataset using a more stringent SNP filter (i.e., allowing no more than 30% missing data), to ensure that our overall results were not influenced by our filtering methods. We additionally thinned sites to one per GBS locus (‐‐thin 90) to reduce linkage disequilibrium among loci. After removing individuals with low coverage at these sites (>80% missing genotype cells), we used this dataset—a matrix of estimated genotype at each site for each individual fish—to generate a genotype covariance matrix of similarity among individuals. The genotype covariance matrix contains a single value for each pair of individuals, or the covariance calculated from all shared loci between that pair of individuals (i.e., loci for which both individuals have a genotype call). We then performed a principal components analysis (prcomp in R) on the genotype covariance matrix.

Upon noticing that differentiation on the fourth principal component corresponded precisely with sex of six individuals of known sex, we further investigated differentiation among these groups which we inferred to be males and females. We conducted discriminant analysis of principal components (DAPC) in adegenet (v2.1.1; Jombart & Ahmed, 2011) in R to identify loci with high loadings explaining differentiation between putative sex groups. We used cross‐validation (xvalDapc) to determine the appropriate number of principal component axes to retain in DAPC analyses and kept the number of PC axes with the lowest root mean squared error. We then calculated heterozygosity in each group independently at loci with high loadings explaining sex differentiation.

We then performed population genomic analyses, including calculating genetic diversity within and divergence between char in different lake groups, on the aligned BAM files using ANGSD (v 0.931; Korneliussen et al., 2014), again using the Atlantic salmon genome as a reference (Lien et al., 2016) and omitting chromosomes associated with sex differences according to our DAPC results. Methods employed in ANGSD take genotype uncertainty into account instead of basing analyses on called genotypes, which is especially useful for low‐ and medium‐depth genomic data (Korneliussen et al., 2014), such as those obtained using genotyping‐by‐sequencing methods. While our data are not extremely low coverage (Buerkle & Gompert, 2013), genotype likelihood methods have the advantage of accounting for differences in depth among sites and individuals, axes of variation for all next‐generation sequencing datasets. From these alignment files, we first calculated the site allele frequency likelihoods based on individual genotype likelihoods assuming Hardy–Weinberg equilibrium (option ‐doSaf 1) using the SAMtools model (option ‐GL 1), with major and minor alleles inferred from genotype likelihoods (option ‐doMajorMinor 1) and allele frequencies estimated according to the major allele (option ‐doMaf 2). We filtered sites for a minimum read depth of 1 and maximum depth of 100, minimum mapping quality of 20, and minimum quality (q‐score) of 20. From the site allele frequency spectrum, we then calculated the maximum‐likelihood estimate of the folded site frequency spectrum (SFS) using the ANGSD realSFS program. The folded SFS was used to calculate per‐site theta statistics and genome‐wide summary statistics, including genetic diversity, using the ANGSD thetaStat program (Korneliussen et al., 2013). We performed each of these steps on all individuals together and then individually for each lake group. We then calculated genetic differentiation (*F*_ST_) between pairs of lakes and pairs of lake groups using the Reich‐Patterson *F*_ST_ estimator (Reich et al., 2009), which is unbiased even for small sample sizes, and estimated 95% confidence intervals for these *F*_ST_ estimates using 100 bootstrap replicates.

We divided individual fish into lake groups and re‐identified and filtered variable sites within each of these groups using VCFTOOLS, so that SNPs retained are only those that are variable within the lake group. With these subsets of individuals, we again generated genotype covariance matrices of individuals and performed principal components analyses. We then analyzed the relationship between the first two principal component axes and fish length within each of the groups of lakes and within each individual lake. We also calculated pairwise relatedness between all individuals within each lake using the gl.grm() function from the package dartR (v1.1.11, Gruber et al., 2018) in R, which calculates the additive relationship matrix using a normalization constant, as described in Endelman and Jannink (2012). If a population is not panmictic, and then, we expect to see a bimodal distribution of pairwise relatedness estimates; otherwise, a unimodal relationship is expected. We further calculated expected heterozygosity, observed heterozygosity, and the inbreeding coefficient (F_IS_) for each lake using dartR. Finally, we conducted DAPC for each lake without assigning group membership a priori. To do this, we first used find.clusters in adegenet to assign individuals to three groups (for Fog3) and two groups (for LTER348). We then conducted a DAPC using these group memberships, optimizing the number of principal component axes to retain by using the optimal α‐score, which minimizes overfitting (Jombart et al., 2010). With these DAPC results, we examined the posterior assignment accuracy of individuals to groups to understand how distinct the phenotypic size classes are genetically.

To formally test whether multiple genetically distinct groups exist within each lake with a bimodal size distribution (LTER348 and Fog3), we used three different programs for ancestry inference: (a) the Bayesian genetic clustering program entropy (Gompert et al., 2014); (b) the maximum‐likelihood ancestry estimation program ADMIXTURE (v1.9, Alexander et al., 2009); and (c) the mixture model‐based individual clustering package stockR (v1.0.74, Foster et al., 2018). Entropy is a program and model much like STRUCTURE (Falush et al. 2003; Pritchard et al. 2000), which also requires no a priori assumption of individual assignment and only requires specification of the number of genetic clusters (K). In addition, entropy incorporates uncertainty about individuals’ true genotypes by taking into account genotype likelihoods as input. Thus, the model integrates outcomes over genotype uncertainty. For each of the two lakes, we ran entropy and ADMIXTURE for K = 1 to K = 5 after removing chromosomes containing putative sex loci. In entropy, for each value of K, we ran three independent MCMC chains of 80,000 total steps, discarding the first 10,000 steps as burn‐in and retaining every 10th value (thin = 10), resulting in 7,000 samples from the posterior distribution of each chain. We checked MCMC chains for mixing and convergence of parameter estimates by plotting a trace of the MCMC steps. We then calculated deviance information criterion (DIC) for each value of K and used these to assess which model fit the structure in our data the best, preferring models with the lowest DIC values. In ADMIXTURE, we ran the model for each value of K and inferred the value of K with the lowest 10‐fold cross‐validation error to be the best fitting number of groups. In stockR, we assigned individuals within each of the two lakes to K = 2 stocks, using 100 bootstrap replicates to assess uncertainty in stock assignments, and compared these stock assignments with the morphological size classes.

## 3 RESULTS

From May 2016 to May 2017, we sampled and photodocumented 233 arctic char including 116 from the “closed” Fog lakes and 117 from the “leaky” LTER lakes (Table 2). Notably, arctic char populations in these lakes vary greatly in size structure (Figure 3) and the lakes form a natural gradient of abiotic and biotic characteristics (e.g., Secchi depth; see Table 1). Arctic char were generally larger in the LTER lakes relative to the Fog Lakes (448.6 ± 8.1 versus. 269.2 ± 9.4; mean total length (mm) ± SE). Despite these distinct size differences across lake types, we did not detect the presence of distinct morphs from the nine measured traits across all size‐corrected morphological data using model‐based clustering (BIC = 8,632.8; ΔBIC > 25 over models with more clusters). Two lakes exhibited bimodal size distributions (Fog3 and LTER348); however, within the lakes we did not identify separate morphometric classifications using cluster analyses (Fog3 BIC = 2,912.3, ΔBIC > 8 over models with more clusters; LTER348 BIC = 2,295.1, ΔBIC > 18 over models with more clusters).

**TABLE 2:** Summary of fish captured and measured for morphological traits during 2016–2017 from study lakes on the North Slope, Alaska. Small, medium, and large size classes were determined via model‐based clustering of raw morphometric trait measurements

	*n*	Mean TL ± SE	Range	Small			Medium			Large		
				*n*	Mean TL ± SE	Range	*n*	Mean TL ± SE	Range	*n*	Mean TL ± SE	Range
All char	233	359.3 ± 8.5	117–601	47	269.2 ± 9.4	117–457	146	375.6 ± 5.8	192–543	40	534.3 ± 7.7	424–601
“Closed” lakes	116	269.2 ± 9.4	117–457	47	269.2 ± 9.4	117–210	64	335.8 ± 6.5	192–436	5	444.0 ± 6.9	424–457
Fog1	19	343.8 ± 10.4	265–453	—	—	—	18	337.7 ± 8.9	265–400	1	—	453
Fog2	2	253.5 ± 103.5	150–357	1	—	150	1	—	357	—	—	—
Fog3	77	232.0 ± 11.1	117–436	45	159.0 ± 2.9	117–210	30	328.5 ± 10.9	192–436	2	427.5 ± 3.5	424–431
Fog5	18	351.4 ± 16.1	209–457	1	—	209	15	346.9 ± 13.2	212–397	2	455.0 ± 1.0	455–457
“Leaky” lakes	117	448.6 ± 8.1	223–601	—	—	—	82	406.6 ± 7.3	223–543	35	547.2 ± 6.1	432–601
LTER345	29	485.4 ± 12.4	304–590	—	—	—	14	439.1 ± 15.4	304–497	15	528.7 ± 10.4	432–590
LTER347	24	447.2 ± 16.9	223–570	—	—	—	20	427.8 ± 16.9	223–543	4	544.5 ± 12.3	522–570
LTER348	64	432.5 ± 11.6	260–601	—	—	—	48	388.3 ± 8.5	260–532	16	565.2 ± 6.2	518–601

**FIGURE 4:**
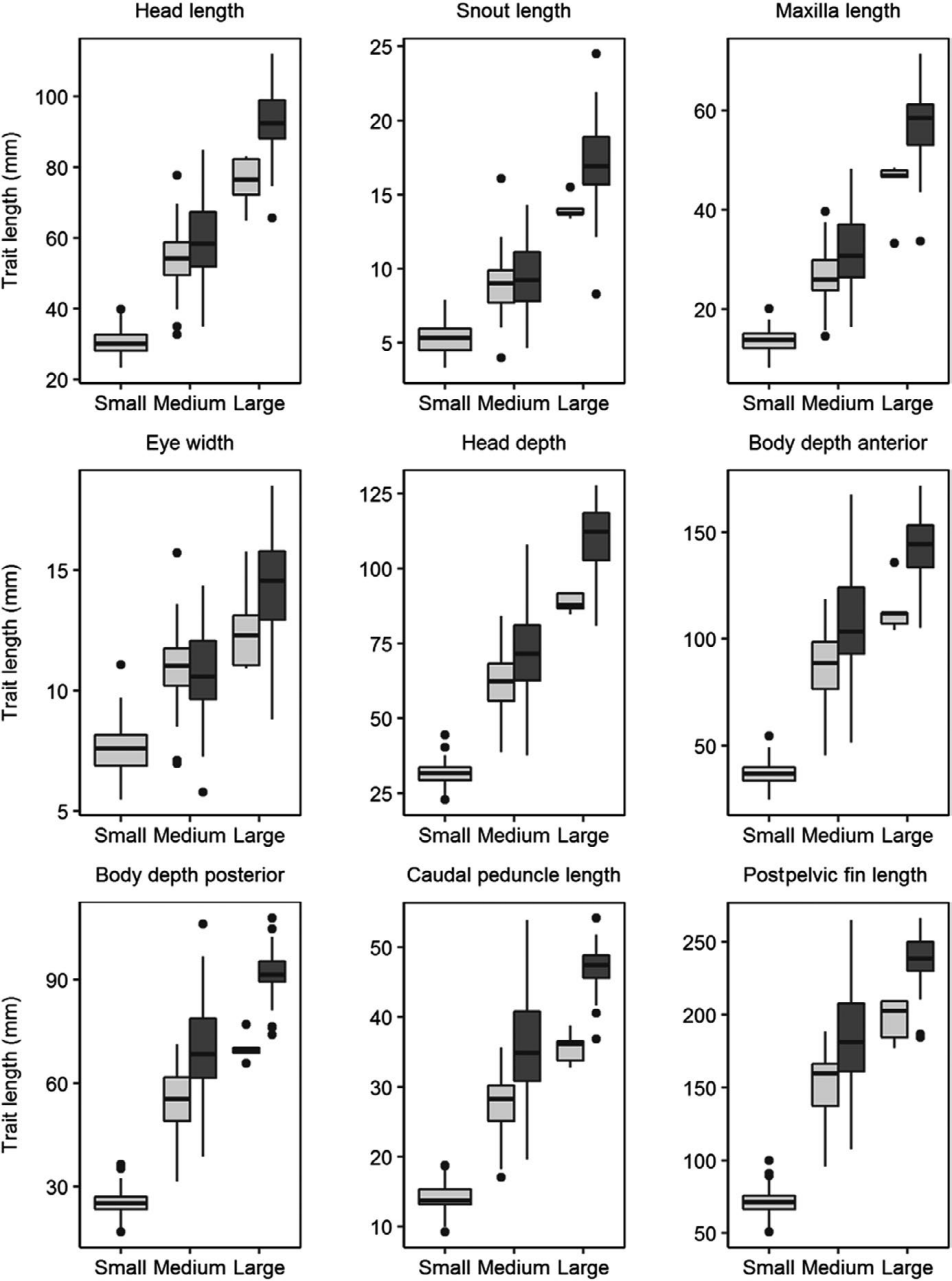
Uncorrected morphological traits measured for arctic char in Fog (light gray) and LTER lakes (dark gray), 2016–2017. *Note*: No char from the LTER lakes clustered into the “small” size class

Following these analyses, based on raw morphological data not corrected for allometry, we detected three distinct size classes across all fish sampled using model‐based clustering (e.g., “small,” “medium,” “large”; BIC = 11,199.7, ΔBIC > 218 over models with fewer clusters; Table 2). Accordingly, all morphometric trait measurements scaled with size class, and in general, morphological traits for fish from LTER lakes for each size class were larger than those in the Fog lakes (Figure 4). All of the small size class fish were found in the Fog lakes, and nearly all (*n* = 45 of 47) came from Lake Fog3.

Arctic char size structure appeared to be influenced more by biotic factors than abiotic factors (Table 3). We observed a significant multivariate effect of Secchi depth (*p* = 0.006), a proxy for primary productivity, and fish density (*p* = 0.005), followed by lake group (*p* = 0.010) in our PERMANOVA of char size classes across lake ecosystems. When tested as the primary predictor, lake group was not significant (*p* = 0.268); however, this predictor (e.g., as a random effect) was significant when coupled with the other predictors of Secchi depth and fish density. No single abiotic predictor was significant (Table 3).

**TABLE 3:** Summary statistics of PERMANOVA analyses to determine best predictors of arctic char size structure across the Fog and LTER lake groups on the North Slope, Alaska, for arctic char captured 2016–2017. Significance codes: (*) *p* < 0.10, (**) *p* < 0.05, (***) *p* < 0.01. For models including more than a single predictor, only the best model's statistics are displayed

Source of variation	*df*	SS	*F*	*p*
Lake group	1	0.22	1.25	0.268
Char abundance	1	0.21	1.15	0.343
Char density by area (fish·ha^−1^)	1	0.18	0.96	0.495
Char density by volume (fish·m^−3^.10^–5^)	1	0.11	0.54	0.809
Secchi depth (m)	1	0.32	2.00	0.065*
Maximum lake depth (m)	1	0.01	0.03	0.997
Mean lake depth (m)	1	0.02	0.07	0.990
Lake surface area (ha)	1	0.10	0.48	0.646
Proportion of lake area < 3 m depth	1	0.08	0.40	0.854
Proportion of lake volume < 3 m depth	1	0.04	0.17	0.985
Lake volume (m^−3^.10^–5^)	1	0.09	0.44	0.577
Secchi depth + Char density by area	2			
Secchi depth	1	0.53	5.15	0.031**
Char density by area	1	0.39	3.79	0.045**
Residuals	4	0.41	—	—
Secchi depth + Char density by area + Lake group	3			
Secchi depth	1	0.28	5.10	0.006***
Char density by area	1	0.43	7.99	0.005***
Lake group	1	0.25	4.57	0.010***
Residuals	3	0.16	—	—

**FIGURE 5:**
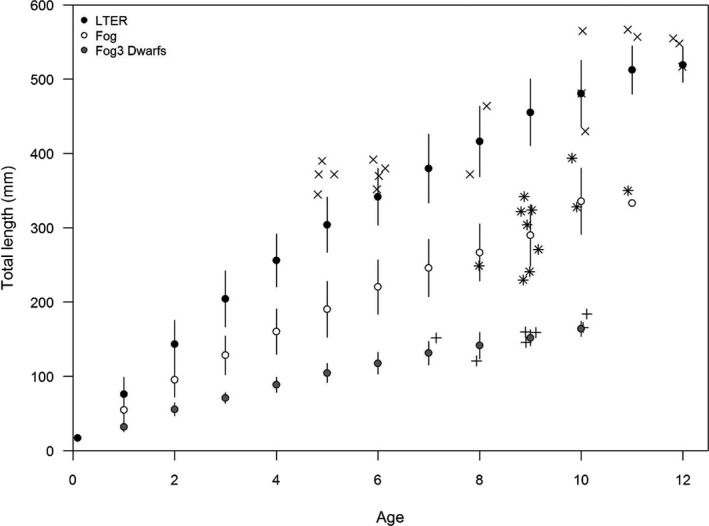
Back‐calculated size at age (mm ± *SD*) for arctic char in Fog and LTER lakes. Fog (white circles) includes model‐based size classes of “medium” and “large” (*n* = 11). Fog3 Dwarfs (gray circles) are the “small” size class and only found in Lake Fog3 (*n* = 7). LTER lakes include “medium” and “large” size classes (*n* = 18). Raw length at age data is represented by symbols (LTER = x, Fog = *, Fog3 Dwarfs = +)

Accordingly, further analyses indicated size and growth differences between lake groups. In the Fog lakes only, the average size of arctic char in the small size class was 159.8 mm (range = 117–210 mm). Despite not clustering as a distinct morph based on size‐corrected morphology or genetic differentiation, our analyses of growth using otoliths indicated “small” char, found almost exclusively in Lake Fog3, and exhibit significantly slower growth rates and smaller size‐at‐age relative to other char in Fog3 and all other lakes (Figure 5). In both the medium and large size classes, mean length of arctic char was significantly larger in the LTER lakes (mean TL of medium char = 406.6 mm, range = 223–543 mm; mean TL of large char = 547.2 mm, range = 432–601 mm; *t* = 23.85, *df* = 91.2, *p* < 0.0001) relative to the Fog lakes (mean TL of medium char = 335.8 mm, range = 192–436 mm; mean TL of large char = 444 mm, range = 424–457 mm; *t* = 7.26, *df* = 144, *p* < 0.0001; Table 2). The larger sizes of arctic char in the LTER lakes were further supported by larger size at age and increased growth rates when compared to the Fog lakes (Figure 5).

Genotyping by sequencing resulted in 382,537,258 150 base pair reads (average 1,015,270 reads per individual), of which 90.3% assembled uniquely to the Atlantic salmon genome. After removing individuals with fewer than 10,000 assembled reads, 5,222,819 variable sites were identified in the complete dataset for 263 individuals. These variants were then filtered to retain only biallelic SNPs more than 90 bp apart, sites with less than 50% missing data, and sites with a minor allele frequency greater than 0.01, resulting in a final genomic dataset composed of 15,934 SNPs for all lakes in both lake groups.

**FIGURE 6:**
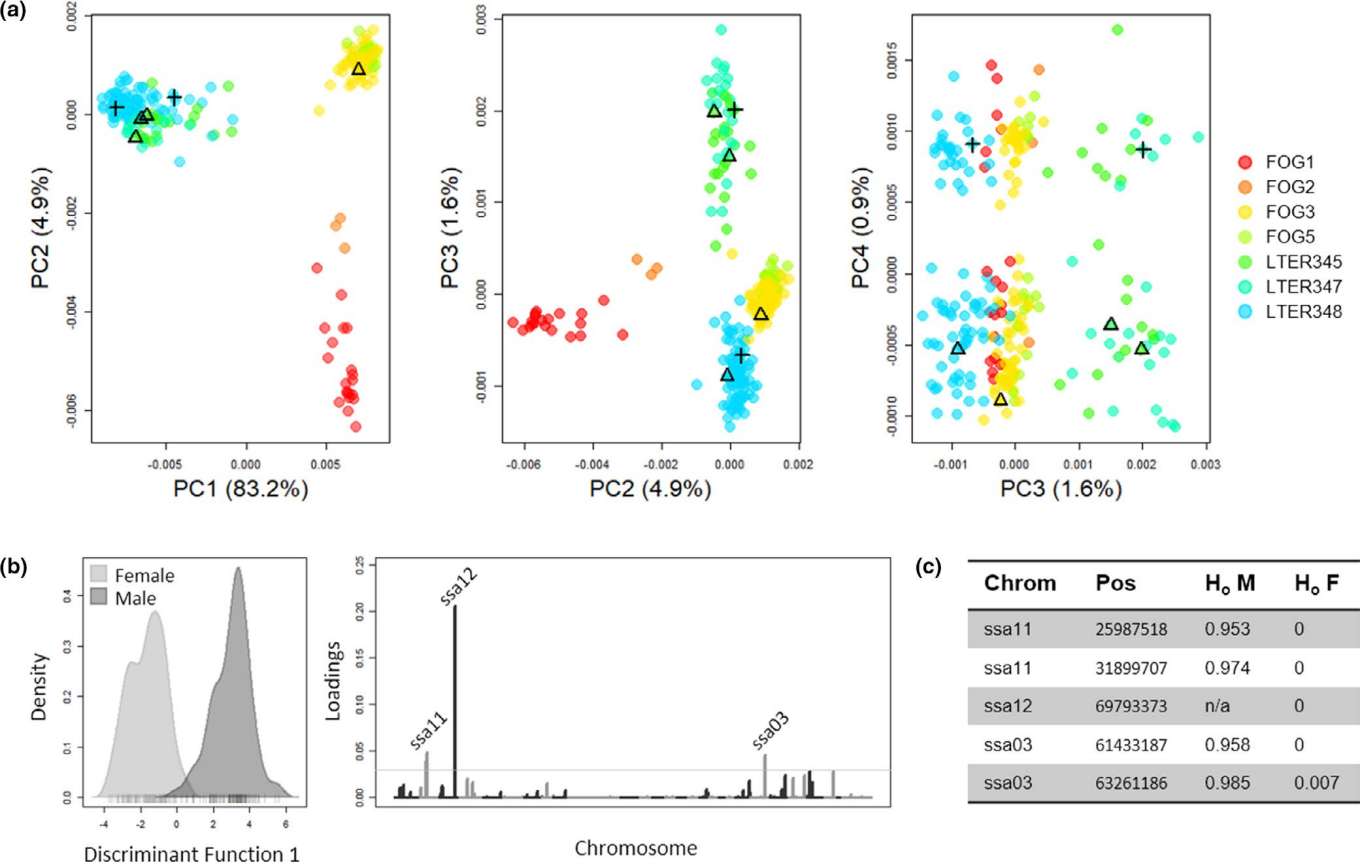
Principal component analysis of genetic data for Arctic char on the North Slope, Alaska. (a) All individuals collected, colored by lake of origin; Fog lakes are in warm colors, and LTER lakes are in cool colors. The first principal component separates Fog and LTER lake groups, the second differentiates lakes within the Fog group, and the third differentiates lakes within the LTER group. The fourth principal component differentiates males and females, thereby indicating genomic differentiation based on sex; individuals with known sex are indicated with symbols (+ = male, triangle = female). (b) Discriminant function analyses clearly differentiate males and females, and three loci in the dataset have high loadings in causing this differentiation. (c) Five SNPs underlie the differentiation at three loci which appear to be sex‐linked. Four of these loci show typical patterns of high heterozygosity in males and no heterozygosity in females, consistent with males being the heterogametic sex. One locus (on ssa12) does not have genotypes in any male fish

Differentiation along the fourth principal component corresponded precisely with sex of six individuals of known sex (Reich‐Patterson *F*_ST_ between sexes = 0.008). Using a DAPC approach, we identified five significant SNPs with strong sex biases (Figure 6b,c). Four of these five SNPs (mapped to Atlantic Salmon chromosomes ssa11 and ssa03) have greater than 95% heterozygosity in the male group and are almost entirely monomorphic in the female group (Figure 6c), consistent with males being the heterogametic sex. One locus has genotypes only in females and not in males (located on Atlantic salmon chromosome ssa12). We removed chromosomes ssa11, ssa03, and ssa12 in further analyses assessing population differentiation and structure.

**FIGURE 7:**
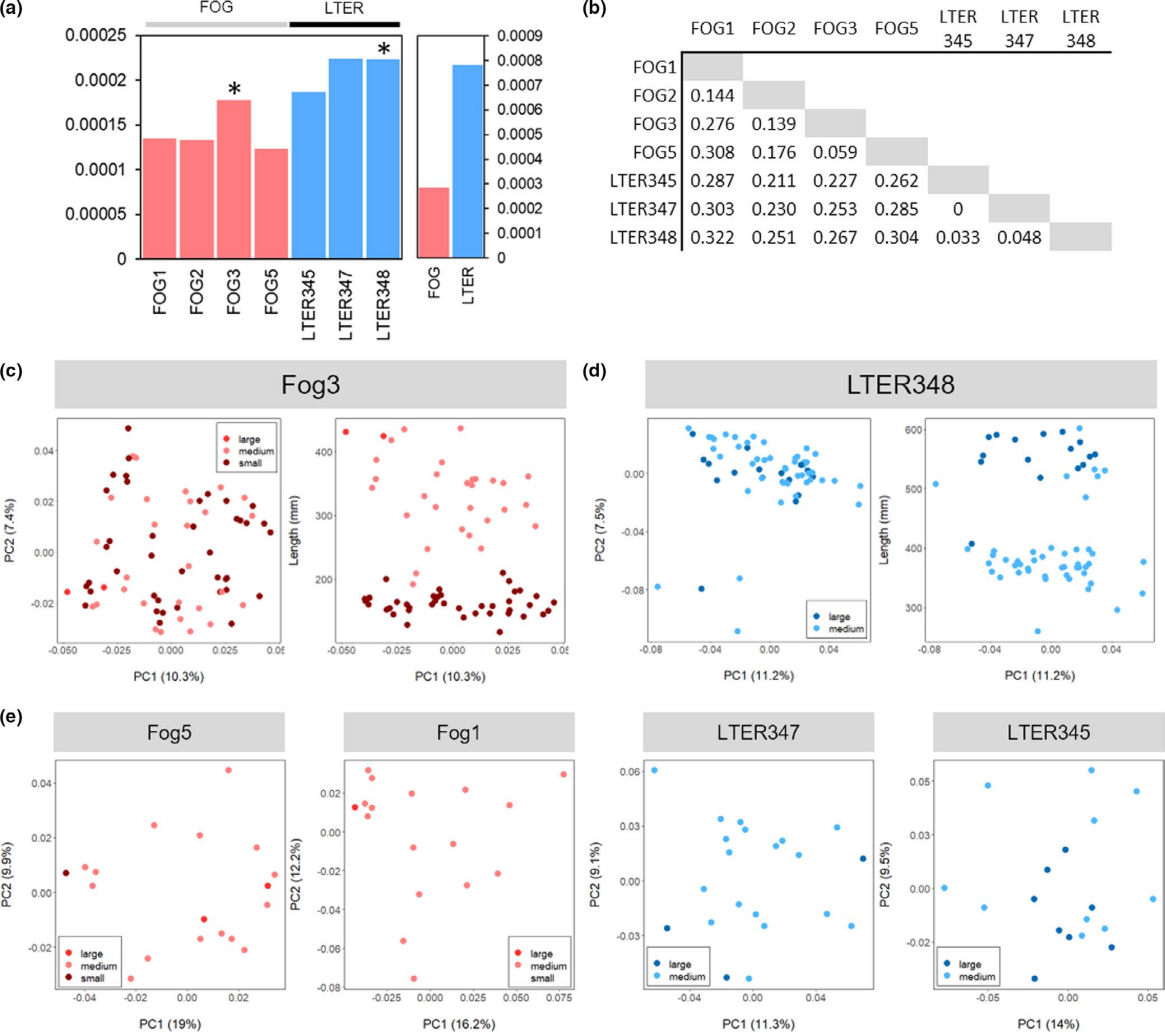
(a) Genetic diversity within each lake sampled (left) and lake group (right), colored by lake group, with asterisks denoting lakes where we observed bimodal size distributions. (b) Differentiation (Reich‐Patterson *F*_ST_) between pairs of lake groups. (c) PCA of genetic data for Lake Fog3 individually, with points colored by size class (small, dark red; large, light red). (d) PCA of genetic data for Lake LTER348 individually, with points colored by size class (small, dark blue; large, light blue). Note that for the right panel in both (c) and (d), PC2 is replaced by total fish length (mm). (e) PCA for individual lakes Fog5, Fog1, LTER 347, LTER 345

Using the dataset omitting chromosomes containing SNPs associated with sex, the char populations in different lake groups were genetically differentiated (mean pairwise weighted *F*_ST_ = 0.208; Figures 6a and 7a,b). Genetic diversity, as measured using Watterson's theta estimator (Watterson, 1975), was almost three times greater in the “leaky” LTER lakes (Θ_w_ = 0.00078) than in the “closed” Fog lakes (Θ_w_ = 0.00029), and both of the lakes exhibiting bimodal size distributions have higher diversity than the other lakes in their respective groups (Figure 7a). All lakes and lake groups have negative inbreeding coefficients (Table S1), suggesting an excess of heterozygotes. Mean pairwise relatedness between individuals did not differ between Fog lakes (two‐sample *t* test, *p* > 0.05; Figure S1, Table S1), but was higher in LTER348 than in the other LTER lakes (two‐sample *t* test, *p* « 0.01; Figure S1, Table S1).

We did not observe genetic differentiation between size classes within lakes with bimodal size distributions (Figure 7). For the two lakes exhibiting a bimodal size distribution of fish, Fog3 (*n* = 79) and LTER348 (*n* = 85), we identified differentiation among individuals within each lake based on sex (Figure 6), but genetic differentiation as indicated by principal component analysis of genetic variation did not correspond to the size of individuals (Figure 7c,d). Likewise, genetic variation did not correspond to size of individuals in the other study lakes (Figure 7e). We found the same patterns when using a data set allowing for only 30% missing data per SNP (8,581 SNPs; Figure S2). When conducting DAPC comparing group assignments to morphological size classes, posterior assignment accuracy was low for both lakes (37.2% in Fog3, 65.1% in LTER348; Figure S3).

To formally test for population structure within the two lakes with bimodal size distributions (LTER348 and Fog3), we first used the Bayesian hierarchical clustering program entropy, choosing values of K (number of clusters) from 1 to 5. The lake‐specific datasets for LTER348 and Fog3 (omitting chromosomes containing sex‐associated loci, and SNPs with > 50% missing data or a minor allele frequency < 0.01) contained 12,730 SNPs for 85 individuals from LTER348 and 10,769 SNPs for 79 individuals from Fog3. For both lakes, the value of K with the lowest DIC value was K = 1 (LTER348 DIC = 1.45 × 10^6^; Fog3 DIC = 1.07 × 10^6^), and groupings for values where K > 1 did not correspond to size structure in either lake (Figure S4). This result was supported by both the ADMIXTURE results (lowest cross‐validation error = 0.225 and 0.231 for Fog3 and LTER348, respectively, at K = 1; Figure S5) and the stockR assessment (no correlation between parent stock and size class; two‐sided Fisher's exact test, *p* = 0.18, 0.075; Figure S6).

## 4 DISCUSSION

While arctic char polymorphism has been widely studied for this Holarctic species in Scandinavian regions (e.g., Klemetsen, 2010; Skúlason, Noakes, et al., 1989), Canada and the United Kingdom (Jonsson & Jonsson, 2001; Recknagel et al., 2017), and even the lower latitudes of Alaska (May‐McNally et al., 2015), little is known about char polymorphism and ecology for populations in the vast number of lakes of arctic Alaska. Here, we set out to quantify the morphological and genetic diversity and divergence of arctic char populations across two contrasting lake complexes in northern Alaska. Although there are clearly different size‐structured populations across lakes, including two individual lakes with strong bimodal size structures, we did not detect differences in size‐corrected morphological traits across or within lakes. Between lake groups at the watershed scale, however, we noted significant differences in genetic structure and length‐frequency distributions, indicating important biotic differences (e.g., primary production, fish density) and the evolutionary distinctiveness of fish in these separated drainages.

Based on age of the glacial landscapes, it is possible that char in the LTER lakes have been present for up to five times longer than populations in the Fog lakes (>53 ka vs. 12–25 ka; Hamilton, 2003). This geologic difference between the lake groups is consistent with the strong genetic differentiation between arctic char populations in the LTER and Fog lakes, and through increased genetic diversity in the LTER lakes compared with the Fog lakes. Despite the older age and higher genetic diversity of the LTER lakes, differentiation between individual lake populations in the Fog lakes is generally stronger, consistent with these lakes being disconnected by surface waters, whereas the LTER lakes are at least partially connected. Further, in other regions with arctic char, lakes of similar ages exhibit genetically distinct populations. In Loch Rannoch, Scotland (~12 ka in age), divergent traits of genetically distinct char were not correlated with the age of lineage divergence; however, Loch Rannoch (surface area = 17 km^2^; maximum depth = 134 m; Bryce et al., 2016) is also much larger than our study lakes. In Iceland, char populations in a series of lakes (~10 ka in age) exhibit varying degrees of phenotypic and genetic differentiation, and divergent morphs are likely due to sympatric divergence (Gislason et al., 1999).

Moreover, ecosystem size has previously been attributed to morphometric differentiation in arctic char (Recknagel et al., 2017), and many lakes where arctic char ecomorphs have been described are large (>10 km^2^) and deep (maximum depth 100 to >200 m) bodies of water (e.g., Arbour et al., 2011; Power et al., 2005; Skoglund et al., 2015). Likewise, May‐McNally et al. (2015) find genetically differentiated ecomorphs only in the largest lake they studied in southwestern Alaska (Lower Tazimina, 520 km^2^), and not the other small lakes in their dataset (0.6–1.2 km^2^). We here studied multiple lakes, singularly and in combination, that are relatively small (<0.3 km^2^) and shallow (generally < 20 m). One possibility for the lack of clear ecomorph formation is that these lakes are not large enough to allow for sympatric ecotype formation. For example, abiotic factors (e.g., lake surface area, maximum depth) were not significant predictors of arctic char size structure in PERMANOVA analyses. As noted, however, biotic factors including primary productivity and arctic char density are more direct contributors to differences in arctic char size structure. Small population sizes, particularly in “closed” systems without substantial or any gene flow from other populations, may limit standing genetic variation available for adaptation (e.g., Schluter & Conte, 2009). In addition, selection is not as efficient in small populations due to the effects of genetic drift, and this may inhibit ecotype formation in very small systems such as those studied here. Comparative work across the northern hemisphere examining the role of lake size in predicting the number of char ecomorphs that form in lakes, and their genomic basis, is warranted to fully understand the ecological and evolutionary drivers underlying the relationship between lake size and char diversity.

Due to the relatively small lakes in this study, biotic factors were more important and char population dynamics in these systems are driven by within‐lake density‐dependent cycles (Budy & Luecke, 2014). Char populations are more abundant in the Fog lakes (Klobucar, 2018; Klobucar et al., 2017) than in the LTER lakes, and these lakes are also generally less productive (Kling et al., 1992). In combination, competition for limited resources could limit the maximum size char can achieve (Downing & Plante, 1993; Naslund et al., 1993; Pechlaner, 1984). In the LTER lakes, it is possible that lake trout and/or burbot have, over time, selected for faster growing individuals and contribute to lower char densities and larger size structure through consumptive effects relative to the Fog lakes where other predators are absent (e.g., Lima, 1998). For example, lake trout and burbot, also present in the LTER lakes, shift to piscivory at a smaller size than arctic char (Kahilainen & Lehtonen, 2003; McDonald & Hershey, 1989), and we rarely observe piscivory (or cannibalism) by arctic char in any of these populations regardless of lake group (Klobucar, 2018; Klobucar & Budy, 2020). The char populations in this study are not exploited by fishing, whereas elsewhere, an increase in char body size often correlates with decreased char population density as a result of fishing harvest (Amundsen, 1989).

Size structure and morphometric differences described for arctic char are often attributed to diverse foraging strategies (e.g., Floro‐Larsen et al., 2016; Malmquist et al., 1992). Water transparency (as an index of primary productivity) and arctic char density were significant predictors of size structure variation between lake groups. That is, in leaky lakes with more primary production, and thus food resources, we observed larger char and a more species‐rich fish community (the LTER lakes). In other work (e.g., Klobucar, 2018; Klobucar & Budy, 2020), we observed generally high trophic overlap (e.g., diet, niche space) between all size classes of char in the Fog lakes. For example, the overlap between estimates of trophic niche space between small and medium sizes class in Lake Fog3 was 98.9%. However, in the LTER lakes, our diet and stable isotope data suggest greater potential for habitat‐related (e.g., littoral versus pelagic) dietary differentiation relative to the Fog lakes. We estimated that trophic niche space of medium char in the LTER lakes with large char in the LTER lakes ranged from 37.2% to 65.7% (Klobucar, 2018; Klobucar & Budy, 2020). Further, in the LTER lakes, we generally observed larger char residing in the littoral zone and consuming increased proportions of littoral prey (e.g., snails), while smaller char appeared to feed more on pelagic prey items and consumed more zooplankton and other aquatic macroinvertebrates (e.g., Trichoptera; Klobucar, 2018). However, at times, across both the Fog lakes and LTER lakes, we surveyed smaller char near shore and larger char offshore via both benthic gill nets and hook‐and‐line sampling. As such, we are confident we sampled across all size classes present within the study lakes.

Overall, we observed only one cluster of char individuals when analyzing all size‐corrected morphological traits, and further, we did not find differences when applying model‐based clustering to head traits (e.g., snout length, eye width, maxilla length, head depth, head length) or body traits (e.g., body depth posterior, body depth anterior, post pelvic fin length, caudal peduncle depth) separately as groups. Previous studies used the same size‐corrected linear measurements as this study to distinguish differences between morphs based on these measurements (e.g., Skoglund et al., 2015). Griffiths (1994) determined that 44% of published size data indicate that arctic char populations were bimodal and included a “normal” and “dwarf” morph within a cohort. While Skoglund et al. (2015) distinguished differences between morphs using some of the same size‐corrected linear measurements we used in this study, they also incorporated geometric measurements into their analyses. In contrast to their study, however, we did not subjectively assign morphs based on appearance or capture location (e.g., littoral versus. pelagic), and used a robust statistical approach used for other fish species to cluster potential morphs (e.g., Muir et al., 2014). Our trophic data (Klobucar, 2018; Klobucar & Budy, 2020) suggest some differentiation of feeding habitats in the LTER lakes, but we still captured char of all sizes in all habitats of the lakes. Thus, we could not reliably assign a potential morphotype based on capture location or phenotypic appearance.

According to our analyses, the size structure observed is not related to genetic structure. If ecotypes were reproductively isolated, we would expect to see genetic differentiation between large and small morphs, an axis of frequent differentiation in arctic char (e.g., Gislason et al., 1999). It is intriguing that the lakes with observed bimodal size distributions have higher genetic diversity than lakes with unimodal size distributions; however, we find that each lake contains one panmictic population (Figures S4 and S5). Although the increased genetic variation does not appear to be due to genetic differentiation between the size classes, it is possible that increased genetic variation facilitates greater size plasticity in these systems, allowing for larger maximum body size. Initially plastic phenotypic responses, such as growth rate variation, may be the first step toward the formation of ecotypes which ultimately become genetically differentiated (e.g., Klemetsen, 2010; West‐Eberhard, 2003, 2005; Woods et al., 2013). Interestingly, in a series of lakes in southwestern Alaska approximately the same age of the Fog lakes (Stilwell & Kaufman, 1996), only one morph was identified in three of four lakes (Woods et al., 2013). However, the lake with two morphs, while also a “closed” system, was a much larger lake (520 km^2^) and these morphs (i.e., “small” and “large”) exhibited different growth rates and were genetically distinct (May‐McNally et al., 2015; Woods et al., 2013). In contrast, in our study, there was no genetic divergence between small and large char in “closed” Lake Fog3, which is smaller than all lakes studied in Woods et al. (2013).

Although genomic data indicated higher genetic diversity in lakes with bimodal size distributions than in those without bimodal distributions, genetic differentiation did not correspond with fish size, and instead was related to genetically based sex differentiation across the dataset. The extent to which sex differentiation is detectable with large genomic datasets is dependent upon the size and differentiation of sex chromosomal regions (Gamble & Zarkower, 2014), and not accounting for sex differentiation can bias analyses of genetic structure when sampling is sex biased (Benestan et al., 2017). For Arctic char, and for salmonids in general, sex is known to be genetically determined (Yano et al., 2013), and previous studies have shown sex differentiation in char genomic datasets (Benestan et al., 2017). Here, we find five SNPs from three loci with strongly sex‐biased patterns, but even this small number of strongly sex‐biased loci causes strong differentiation between the sexes in PCA. Four out of five of markers have high heterozygosity in males and no heterozygosity in females, indicating heterogametic males in these populations, consistent with general patterns known from other salmonids (Yano et al., 2013). Although the loci map to different Atlantic salmon chromosomes (ssa03, ssa11, and ssa12) which are not implicated in sex for Atlantic salmon (ssa02; Lien et al., 2016; Phillips et al., 2009) or in the recent Arctic char genome assembly (AC04q and AC04p.2, which map to Atlantic salmon ssa09; Christensen et al., 2018), we would expect that these loci either fall on the arctic char Y‐chromosome or are linked to sex‐determining loci; salmonid Y‐chromosomes have little conserved synteny across species although they do usually share the master sex determination gene *sdY* (Yano et al., 2013). It is worth noting that we thinned our dataset to include only one SNP per locus, and without this thinning, we may have found a larger number of sex‐linked SNPs; however, as sex differentiation was not the goal of this study, we did not conduct these analyses here. Regardless, the strong differentiation we observe even with a small number of sex‐linked SNPs underscores the importance of accounting for sex in the analysis of genomic datasets, as sex‐linked differentiation could easily be misinterpreted as cryptic genetic structure if sexes were unknown. Once these sex‐linked regions were removed from our dataset, no genetic structure remained, even within lakes with bimodal size distributions (Figure S1).

In this study, we provide some of the first descriptions of arctic char morphological and genetic divergence in lakes of northern Alaska. As lakes in this region are numerous and diverse (e.g., different abiotic and biotic characteristics), we highlight the importance of understanding how the morphological and genetic divergence of different arctic char populations can vary over small spatial scales. From a conservation standpoint, it is important to maintain the diversity of arctic char populations (e.g., populations of varying size structures) across the landscape, in order for continued persistence during periods of rapid change. In the short term, increases in temperature (and thus, production) may increase population density of the char populations studied here, especially in the Fog lakes. However, population increases could actually lead to future population susceptibility, if food does not also increase to meet increased metabolic demand (Budy & Luecke, 2014) or habitat becomes limiting (e.g., thermal conditions). From a subsistence standpoint, it is important to maintain healthy populations and sustainable harvest. While our study lakes are not exploited by harvest, the lakes are representative of thousands of lakes in northern Alaska with arctic char populations. Based on our growth analyses, lakes similar to the LTER lakes could be susceptible to overharvest due to overall char densities and perceived low recruitment. On the other hand, closed lakes with dense populations similar to the Fog lakes could potentially benefit from some harvest, to decrease competition and thereby likely increased mean individual growth, as a density‐dependent response. Continued study of these lakes as climate warming proceeds will provide further insight into these issues and predictions.

Additionally, we show that the size‐structured populations present in some lakes in this area are likely due to plasticity in growth rates rather than genetic differentiation. Given that genetically differentiated ecotypes are frequent in Arctic char, these populations may be constrained in divergence by the environment and species interactions in these lakes, but also represent an interesting case study in how plasticity may function as an initiator of ecotype formation. Predators in the LTER lakes (e.g., lake trout, burbot) likely inhibit the evolution of “dwarf” char in these lakes because these predators would consume smaller individuals. In fact, through minnow trap surveys, we observed no young of year for any species in these lakes (Klobucar and Budy, unpublished data). In the Fog lakes, limited spawning habitat availability may limit genetic divergence or the potential for spawning habitat segregation between ecotypes. Thus, these lakes may present a unique window into the early plasticity‐based stages of ecotype formation in arctic char, yet be constrained in the formation of genetically divergent ecotypes by the small size of these ecosystems and through species interactions with predators. The persistence of arctic char populations across the landscape, especially in a warming climate, may depend both on phenotypic plasticity and genetic diversity that confers adaptive potential, especially as hydrologic cycles and lake connectivity shifts with a changing climate.

## CONFLICT OF INTEREST

None declared.

## AUTHOR CONTRIBUTIONS

**Stephen L. Klobucar:** Conceptualization (lead); Data curation (lead); Formal analysis (lead); Investigation (lead); Methodology (lead); Visualization (lead); Writing‐original draft (lead); Writing‐review & editing (equal). **Jessica A. Rick:** Data curation (supporting); Formal analysis (supporting); Methodology (supporting); Visualization (supporting); Writing‐review & editing (supporting). **Elizabeth G. Mandeville:** Formal analysis (supporting); Methodology (supporting); Writing‐review & editing (supporting). **Catherine E. Wagner:** Conceptualization (supporting); Funding acquisition (supporting); Supervision (supporting); Writing‐review & editing (supporting). **Phaedra Budy:** Conceptualization (equal); Funding acquisition (lead); Investigation (supporting); Project administration (lead); Supervision (lead); Writing‐review & editing (supporting).

## Supporting information

Supplementary Material

## Data Availability

Arctic char morphometric data were deposited in the Dryad Digital Repository, https://doi.org/10.5061/dryad.80gb5mkqc, and char genetic data, https://doi.org/10.5061/dryad.1vhhmgqrs. Raw genetic data are available in NCBI SRA BioProject PRJNA687211.
