# Supplementary material for: Investigating the morphological and genetic divergence of arctic char (Salvelinus alpinus) populations in lakes of arctic Alaska
Source: Ecol Evol. 2021 Mar 5;11(7):3040–57. doi: 10.1002/ece3.7211 (PMC8019052; doi:10.1002/ece3.7211)
Supplement: Supplementary file 1 — Supplementary Material [file ECE3-11-3040-s001.docx]

Supplemental Figures and Tables

**Table S1**. Kinship estimates, observed (H_o_) and expected (H_s_) heterozygosites, and inbreeding coefficient (F_IS_) for each lake in this study. For each value, the mean and standard error are given.

| Lake | Mean Kinship | Mean H_o_ | Mean H_s_ | F_IS_ |
| --- | --- | --- | --- | --- |
|  |  |  |  |  |
| *Fog Lakes* | *-0.005(0.013)* | *0.22(0.03)* | *0.16(0.02)* | *-0.09(0.04)* |
| Fog1 | -0.016(0.004) | 0.41(0.08) | 0.37(0.04) | -0.32(0.09) |
| Fog2 | -0.115(0.014) | 0.74(0.17) | 0.43(0.05) | -0.65(0.24) |
| Fog3 | -0.005(0.002) | 0.27(0.04) | 0.17(0.02) | -0.21(0.04) |
| Fog5 | -0.018(0.009) | 0.40(0.09) | 0.25(0.05) | -0.33(0.10) |
|  |  |  |  |  |
| *LTER Lakes* | *-0.004(0.004)* | *0.20(0.03)* | *0.14(0.01)* | *-0.12(0.03)* |
| LTER345 | -0.018(0.004) | 0.38(0.07) | 0.26(0.04) | -0.28(0.08) |
| LTER347 | -0.024(0.005) | 0.28(0.06) | 0.21(0.04) | -0.18(0.07) |
| LTER348 | -0.005(0.003) | 0.22(0.04) | 0.15(0.02) | -0.15(0.03) |
|  |  |  |  |  |


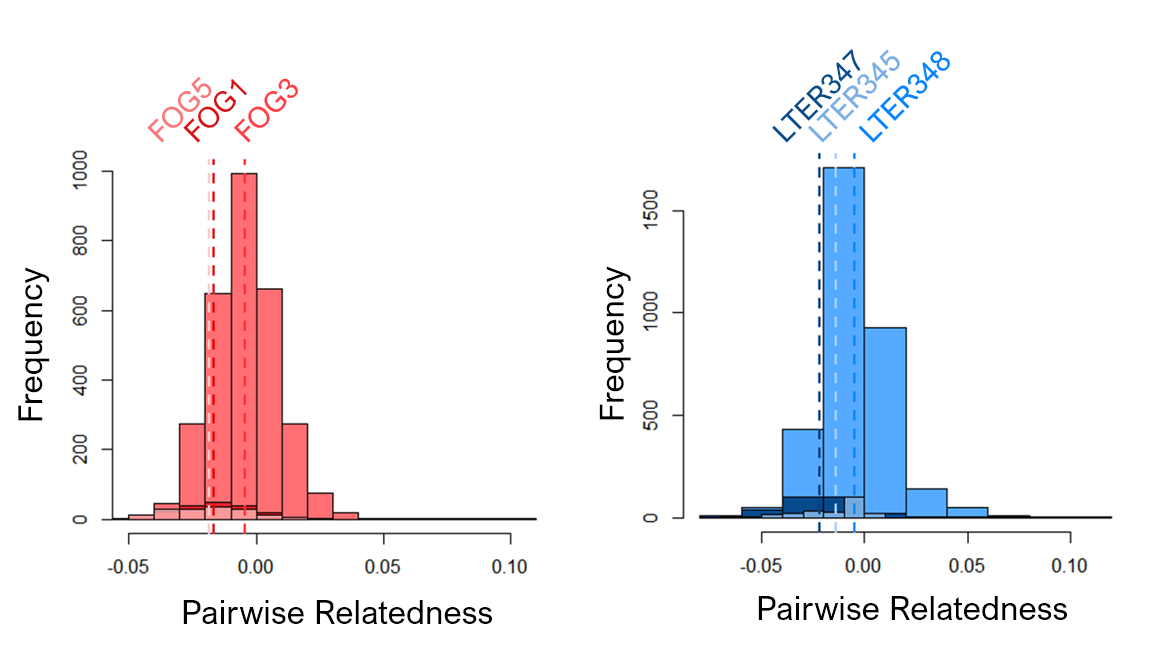


**Figure S1**. Distribution of pairwise relatedness between individuals within each lake in our analysis. Histograms are colored by lake, and average pairwise relatedness for each lake is indicated with a vertical dashed lake. Fog2 was omitted due to low sample size. Mean pairwise relatedness was not significantly different between the Fog lakes but was significantly less negative for LTER348 when compared to LTER345 and LTER347 (two-sample T-test, *p* << 0.01).


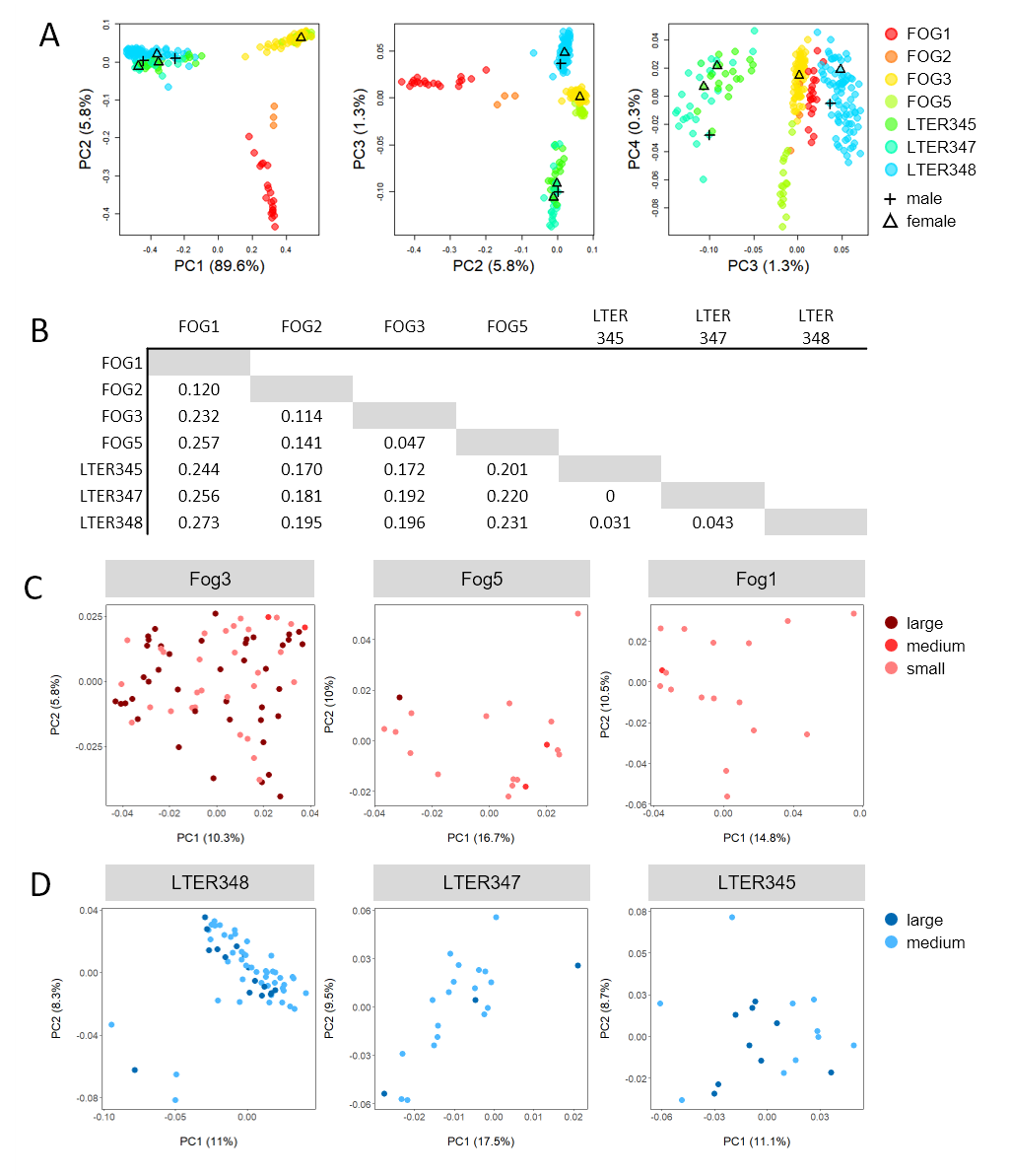


**Figure S2**. Genetic differentiation analyses conducted using the SNP data set allowing for a maximum of 30% missing data per SNP, showing the same general patterns as presented in the main figures. (A) Principal component analysis of all Fog and LTER lakes together, colored by lake and with phenotypically-sexed males and females indicated, as in Figure 6. As with the less stringent data set, PC4 separates the two sexes, but with less distinction than when allowing for 50% missing data. (B) Differentiation (Reich-Patterson F_ST_) between pairs of lake groups. (C) PCA of genetic data for lakes Fog3, Fog 5, and Fog1, with points colored by size class (small, dark red; medium, red; large, light blue). (D) PCA of genetic data for lakes LTER348, LTER347, and LTER345 individually, with points colored by size class (small, dark blue; large, light blue).


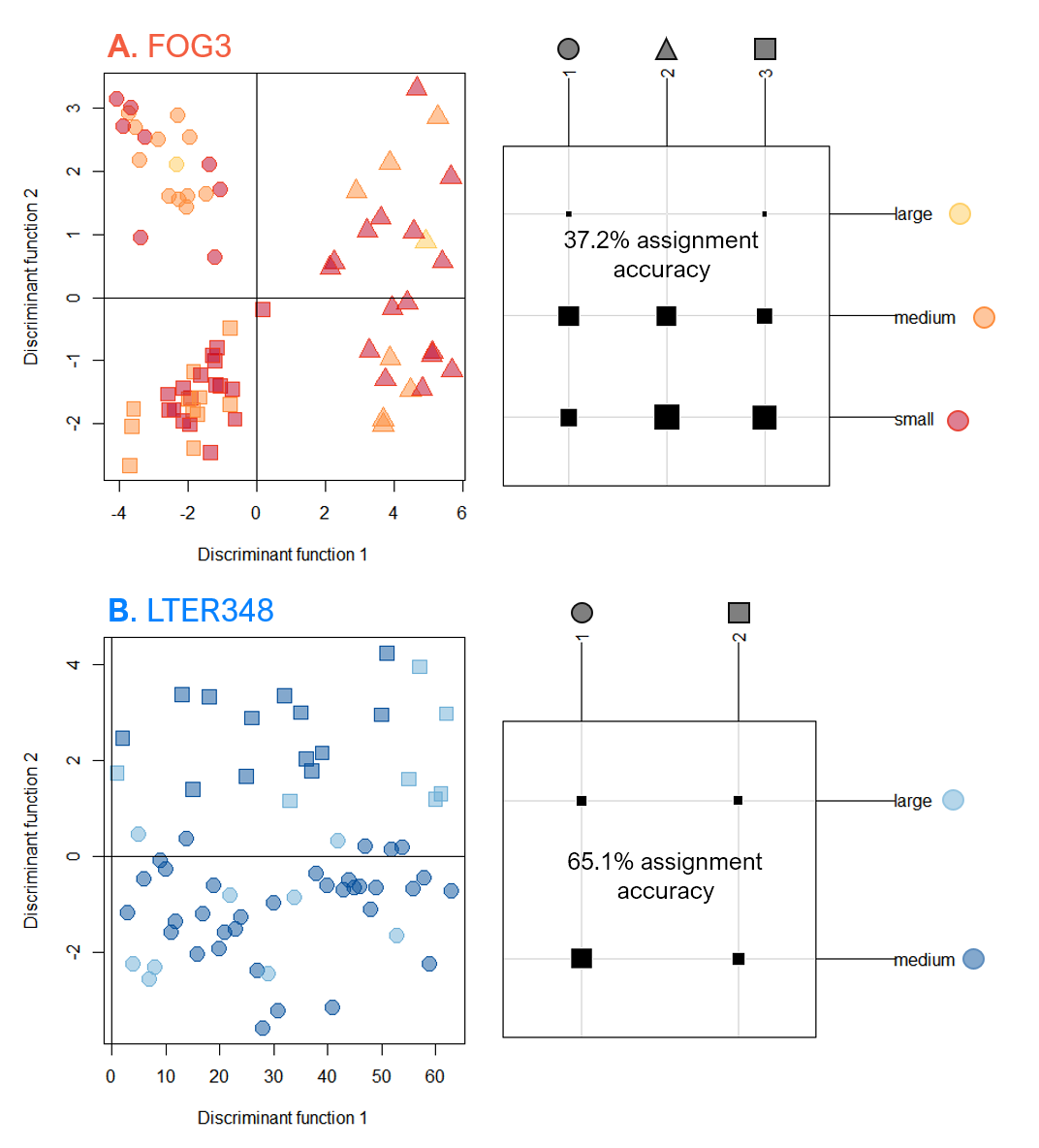


**Figure S3**. DAPC results for the two bimodal lakes. For each lake, the left hand plot shows the distribution of individuals along the first two discriminant function axes, with individuals colored by morphological size class. Shapes indicate the assigned group using kmeans clustering without prior information on group membership (find.clusters() in adegenet). Righthand plots show DAPC posterior assignment accuracy, with DAPC-assigned class along the top and known size class along the righthand side. Overall assignment accuracy is indicated for each lake.


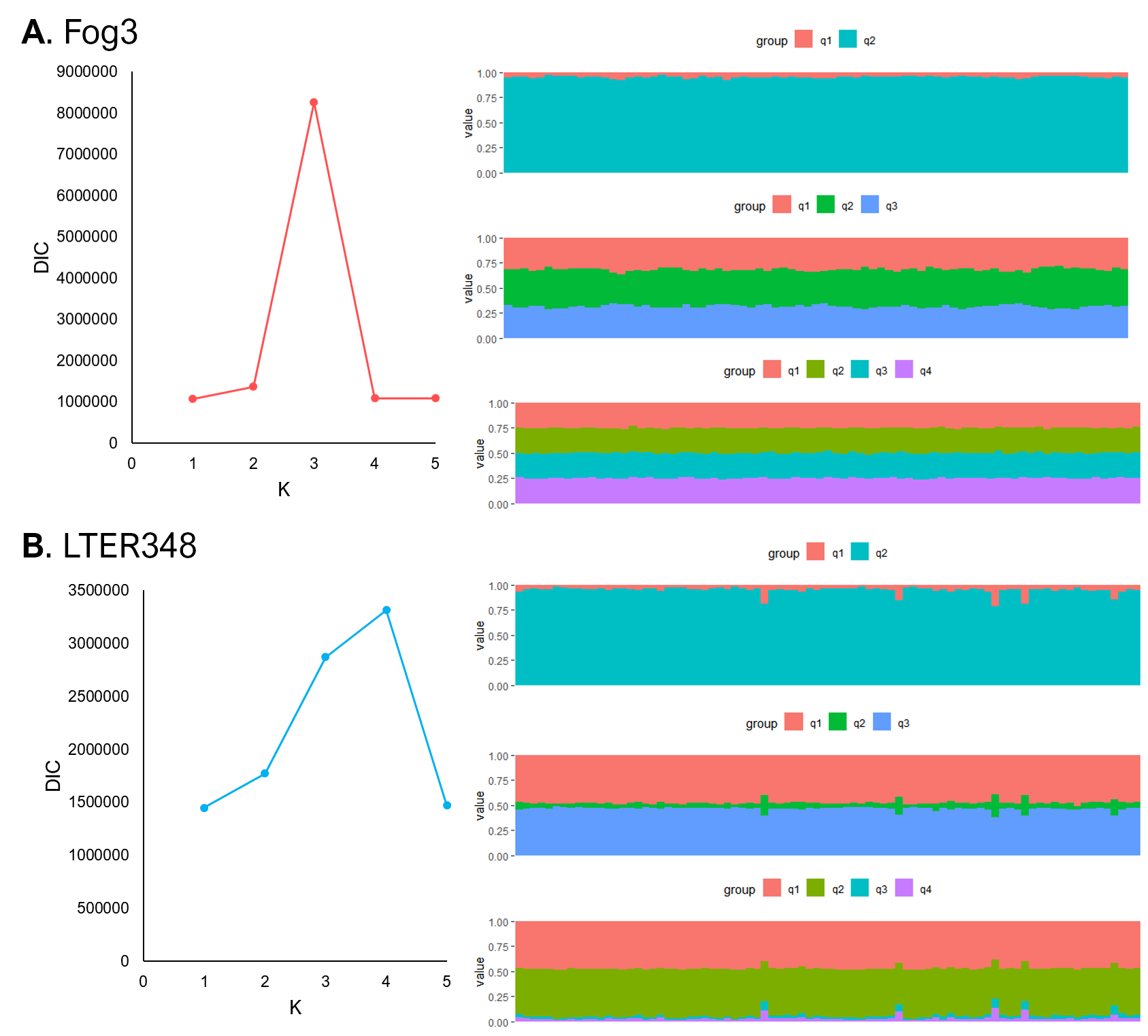


**Figure S4**. Genetic structure analyses in entropy show a lack of genetic differentiation within lakes (A) Fog3 and (B) LTER348. Lefthand plots show deviance information criteria (DIC) for values of K=1 to K=5. The value of K with lowest DIC is the best-fitting model for the data. Righthand plots show proportion of group membership (q) for each individual for K=2 to K=4, with colors indicating different genetic groups.


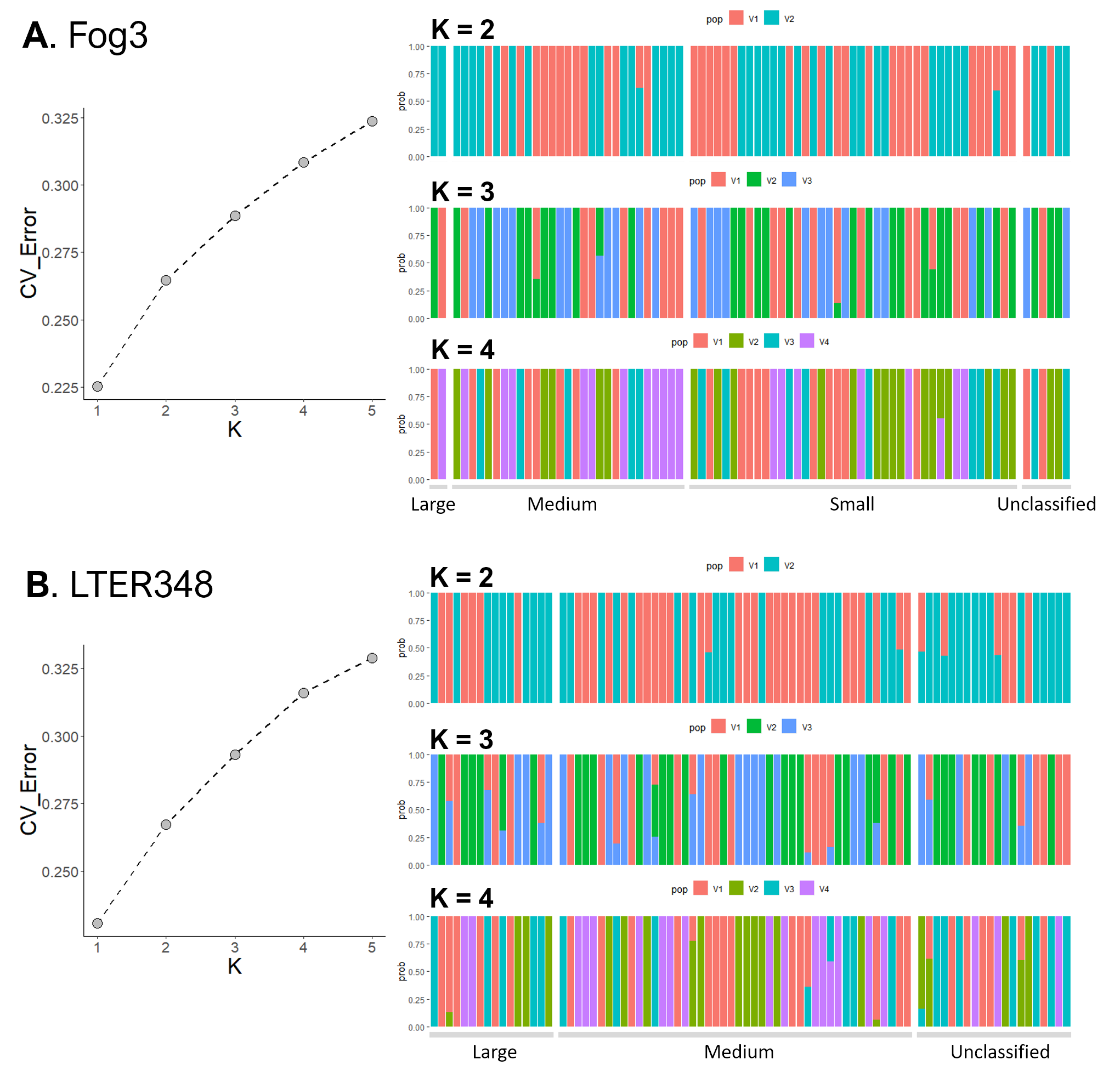


**Figure S5**. Ancestry estimation using ADMIXTURE shows a lack of correspondence between size classes and genetic groups in both (A) Fog3 and (B) LTER348. Lefthand plots show 10-fold cross-validation error for values of K=1 to K=5. The value of K with lowest cross-validation error is the best-fitting model for the data. Righthand plots show proportion of group membership (q) for each individual for K=2 to K=4, with colors indicating different genetic groups.


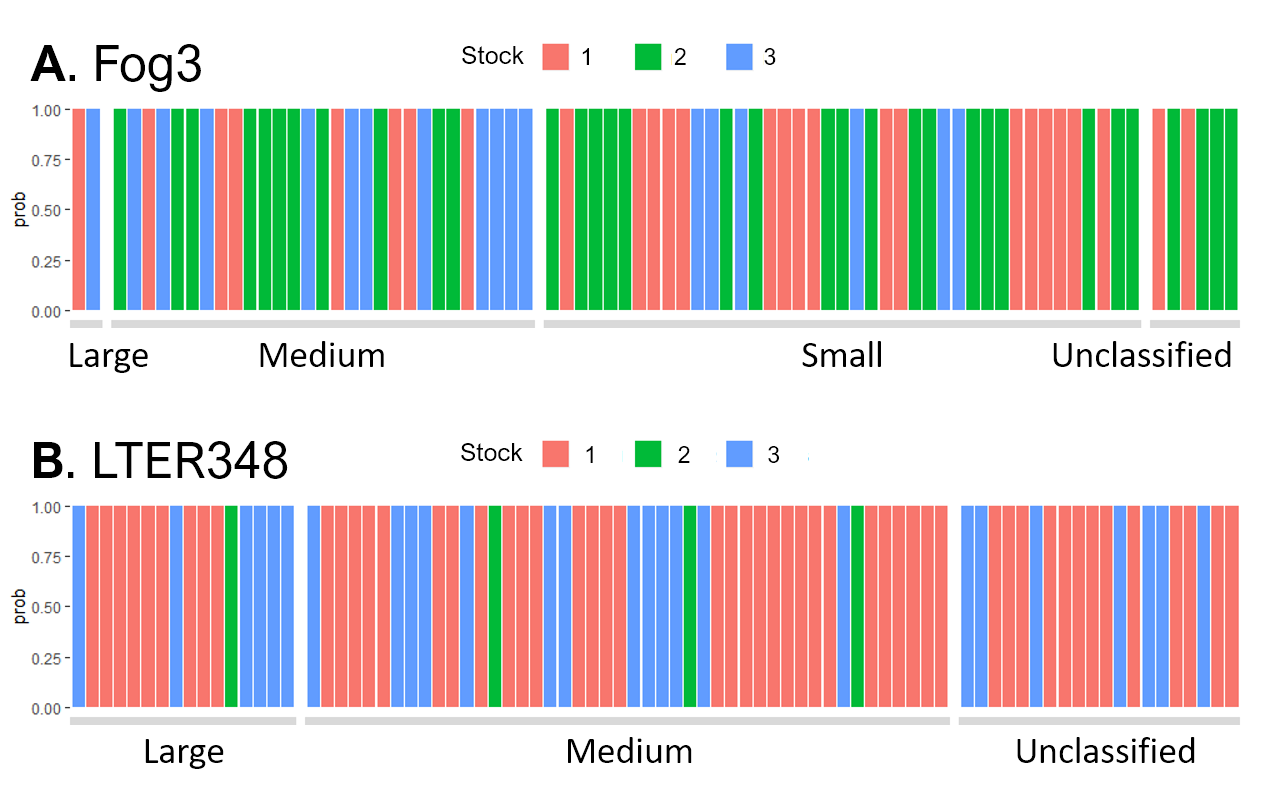


**Figure S6**. Ancestry estimation using stockR shows a lack of correspondence between size classes and genetic groups in both (A) Fog3 and (B) LTER348. Colors correspond to inferred genetic group, and each bar represents one individual. Stock classifications do not correspond with phenotypic size class (two-sided Fisher’s Exact test, *p* = 0.18, 0.075).
